# Understanding exposure risk using soil testing and GIS around an abandoned asbestos mine

**DOI:** 10.5334/aogh.4624

**Published:** 2025-01-22

**Authors:** Abhijeet V. Jadhav, Nilesh Gawde, Ramesh Veerappan, Yeyong Choi, Arthur L. Frank

**Affiliations:** 1 Tata Institute of Social Sciences, Mumbai, India; 2 Asian Citizen’s Center for Environment and Health, Seoul, South Korea; 3 Department of Environmental and Occupational Health, Dornsife School of Public Health, Drexel University, Philadelphia, PA 19104 USA

**Keywords:** Asbestos, chrysotile, mining, pneumoconiosis, environmental exposure, spatial analysis, mesothelioma, asbestosis

## Abstract

*Background:* Abandoned asbestos mines are a potential source of environmental contamination and exposure for nearby residents. The asbestos exposure risk may persist even after the cessation of mining activity if the mine is not properly closed. One such abandoned mine is at Roro Hills in the Jharkhand state of India. There are limited studies examining soil contamination and asbestos exposure to nearby residents due to abandoned mines.

*Objectives:* The aim of this study is to examine the presence of asbestos in the residential areas of villages surrounding an abandoned asbestos mine and to understand the spread of visible asbestos dust using geographic information system map analysis.

*Methods:* This study examined the presence of asbestos in soil samples from four villages surrounding an abandoned asbestos mine using the scanning electron microscopy technique. The study also compared satellite images taken 13 years apart to determine whether the mine waste containing asbestos had spread over time.

*Findings:* The soil sample testing indicated that, out of 16 soil samples from residential areas, 12 showed the presence of chrysotile asbestos. It was found in the map analysis that asbestos-containing areas had enlarged by around 20% in those years.

*Conclusion:* The evidence indicated the presence of asbestos in the soil of nearby residential areas around the mine, and this contamination has spread over the years. Similar studies at other mine locations are needed, and timely interventions are warranted to protect nearby residents.

## Introduction

Asbestos is a fibrous mineral found in the earth’s crust. It has two main forms, serpentine (curly fibers; type: chrysotile) and amphibole (thin and straight needle‑like fibers; types: actinolite, amosite, anthophyllite, crocidolite, and tremolite). Asbestos has many industrial applications because of its extreme resistance to high temperatures and chemicals. It is mined and processed for a wide range of uses. In this process, many miners, workers, and populations residing close to mines and factories are exposed to asbestos. It is a strong carcinogen that can cause cancers of the lung, trachea, larynx, and pleura (mesothelioma) [1]. Asbestos is also fibrogenic to the lungs and can cause fibrotic lung disease called asbestosis [2]. The group of all these diseases is called asbestos‑related diseases (ARDs). According to the World Health Organization (WHO), even the smallest amount of asbestos can cause ARDs, and no amount is safe [2, 3]. It is estimated that around 255,000 people die each year due to ARDs [4].

To date, about 68 countries have banned asbestos after understanding its public health impact [5]. Most of these are industrialized or high‑income countries (HICs) that have understood that asbestos cannot be handled safely. However, the mining, processing, and use of asbestos continues in many other countries, exposing unaware individuals and communities. Asbestos mines are one of the major sources of harmful exposure to asbestos and environmental contamination [6, 7]. Functional as well as closed asbestos mines can pose an exposure risk. These mines can contaminate air, soil, and water with asbestos. If asbestos mines are not closed properly, the waste material generated may continue to contaminate and expose the surroundings [7, 8].

India stopped the renewal of old mining leases in 1986, leading to the stoppage of asbestos mining in 2014, as the lease periods were between 20 and 30 years [9]. There are many abandoned mines without proper closure. Proper mine closure ensures ecological restoration, safe and sustainable waste disposal, structures for safe closure, biodiversity regeneration, and rehabilitation and restoration of communities around the mines from the socio‑economic aspects [10, 11]. In India, the older rules related to mining operations, safety norms, and mine closure were not very stringent, unlike in developed countries. Implementation of these laws was often suboptimal [12]. India’s new National Mineral Policy (2019) highlights the importance of proper mine closure [10]. The ‘Final Mine Closure Plan’ manual (2022), by the Indian Bureau of Mines (IBM), also focuses on sustainable waste disposal [10, 11, 13]. These are recent guidelines, and many old mines were not appropriately closed. People in the vicinity of an old abandoned asbestos mine may be at continuous risk of exposure [7].

Until 2014, many asbestos mines were operational, located mainly in the states of Rajasthan, Andhra Pradesh, and Jharkhand. Until 2001, there were 30 functional asbestos mines in India, and many closed before that [14]. It is crucial to study the presence of asbestos, its spread, and exposure in nearby communities around such abandoned mines [15]. There are limited studies in India that examined the spread of asbestos around such mines, its presence in the surrounding residential areas, and possible exposure. It will help in developing future interventions to protect at‑risk residents.

**Figure 1:**
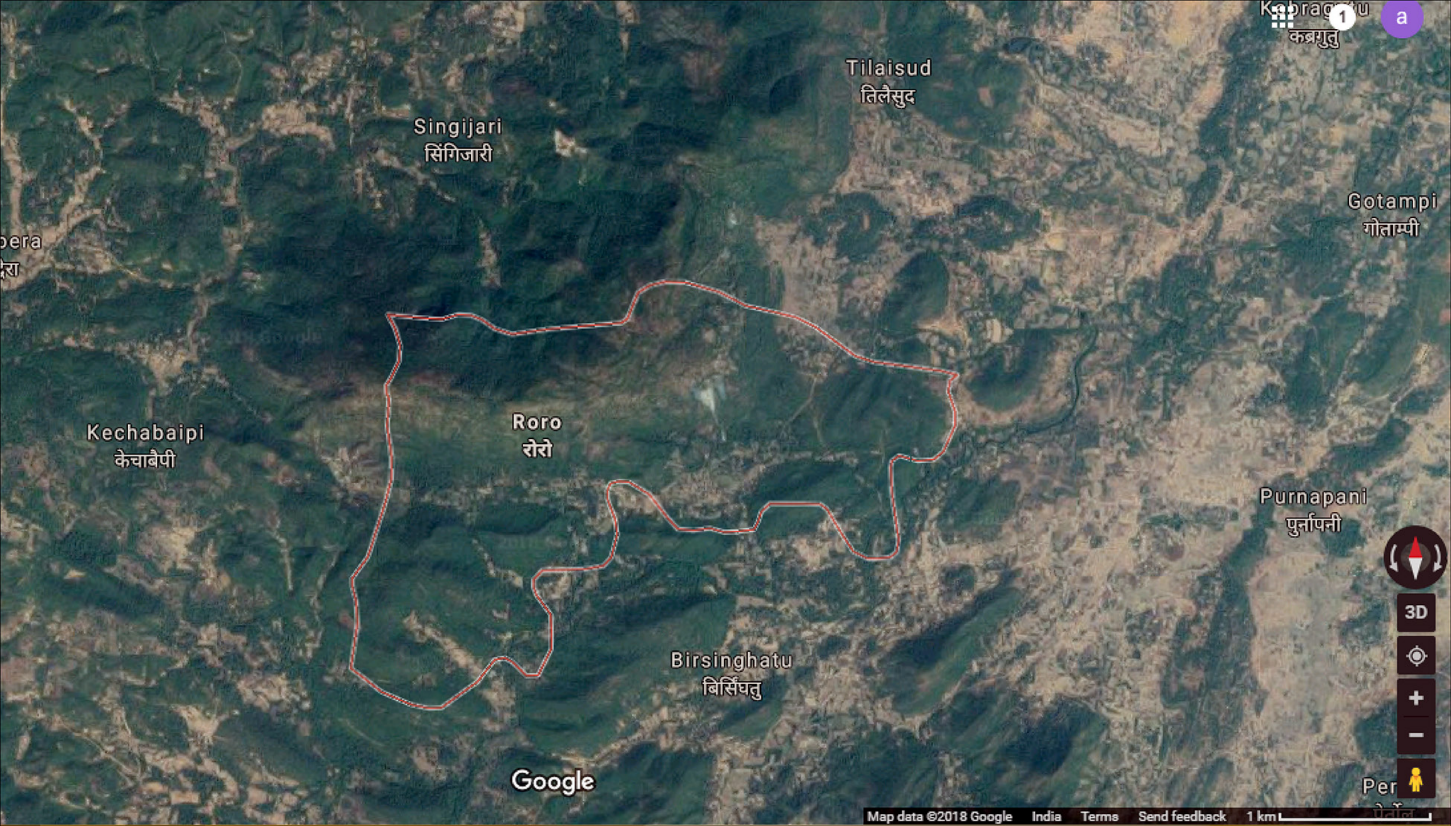
Roro village, with an asbestos pile in the center, and surrounding study villages.

**Figure 2:**
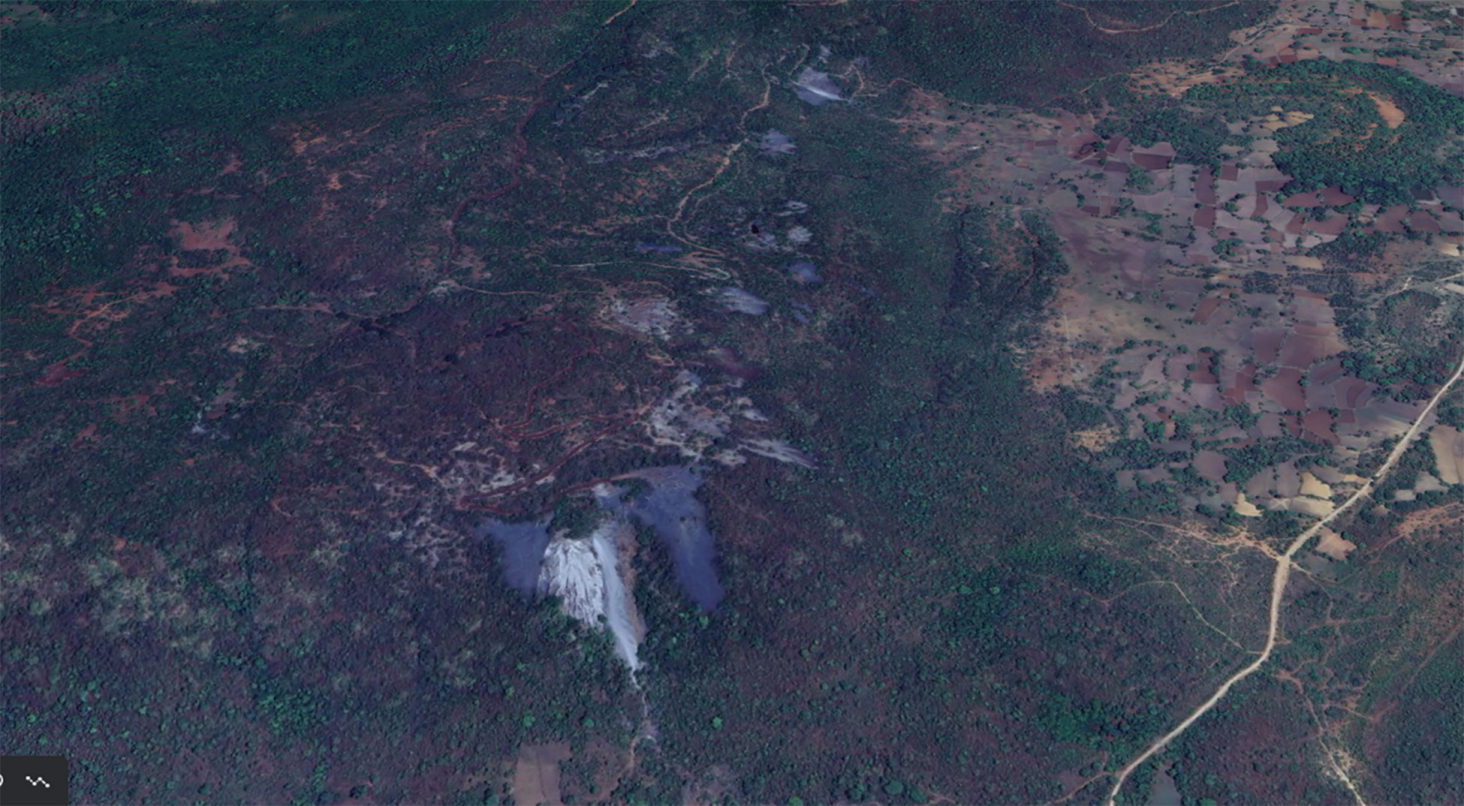
The Roro hill top with mine waste deposits at multiple locations.

The West Singhbhum district of Jharkhand state in India has Roro Hills. It is a rural and forested area. There was an asbestos mine that was operational from 1963 to 1983. Tribal villages surround this hill and the mine. Four study villages—Birsingh Hatu, Roro, Singijari and Tilaisud—are within an aerial distance of three kilometers from the Roro mines, as seen in Figure 1 and Figure 2. According to the 2011 census, the total population of these four villages was 2454. The community living there was tribal and has socioeconomic vulnerabilities and compromised access to healthcare.

An average person is generally unaware of the presence of harmful asbestos in the surroundings or the possible diseases it can cause. The proper closure of mines and minimizing exposure risks from hazardous areas are an environmental health priority, but these warrant empirical evidence when it comes to implementation.

During its operational phase, the asbestos production from the Roro mine was in the range of 0.4–0.5 million tons per year, with around 1500 people working there [12]. One side of the mountain is completely covered with waste material. This material is a remnant of stones from the mine that were processed and from which the asbestos‑rich portion was taken; the rest was tossed on the mountain slopes. Waste still contains asbestos in significant proportions and acts as an exposure source for environmental contamination. The estimated waste material is 0.7 million tons in the main pile of dust [12]. That excludes other smaller piles on the hill where waste was dumped. In the main pile, two distinct parts are visible. One is of stone gravel, and the other is of fine powdery dust. This dust has been spreading with rain and wind for decades. All the villages that were in the immediate vicinity of the mine were selected as study sites. These villages have been exposed for years to asbestos dust. This study aims to detect the presence of asbestos in soil samples from residential areas and to understand its spread in the villages around the Roro mine over the years.

## Materials and Methods

The study has two components. The first one is related to testing soil samples for the presence of asbestos, and the second is conducting spatial analysis using a geographic information system (GIS) to understand the spread of visible asbestos‑contaminated areas over time.

Soil samples were collected from the residential areas of the study villages, using global positioning system (GPS) tagging. From every village, four soil samples were collected, making a total of 16 samples. Superficial soil samples of 3–4 grams were collected in clean polyethylene packets with zip liners using a clean spoon. The packets were given code numbers. The village name, colony/area of the village, and longitude–latitude coordinates were noted along with the soil code number. It was attempted to cover all the residential areas of the villages, and each sample was collected from a different location close to different groups of houses. Each sample was packed separately with one more sealed packet at the location itself. One negative control sample was also added (first in the series of the laboratory reports). All the soil samples were packed and sent to the laboratory by post. The soil sample testing was conducted by the Institute of Specialized Analysis for Asbestos (ISAA) in South Korea, a licensed laboratory with international standards. The laboratory used a scanning electron microscope (SEM)‑based technique, and it gave information about (i) the presence or absence of asbestos, (ii) the type of asbestos in the positively confirmed samples (based upon molecular composition ratio), and (iii) the dimensions of the fiber (long asbestos fiber [LAF] or short asbestos fiber [SAF]) along with a photograph of the fiber. The full report of the SEM examination of all the soil samples provided by the laboratory is in the supplementary files of this article. The fiber is called a long asbestos fiber (LAF) if it has a length of more than 5 microns and has a length–width ratio above 3. Fibers shorter than these dimensions are called short asbestos fibers (SAFs). The LAFs are proposed to be more carcinogenic and fibrogenic [16].

The second part of this study consisted of the GIS spatial analysis. For this purpose, high‑resolution, open‑source GIS images of the study region were acquired. Remote sensing and GIS techniques are highly useful in analyzing temporal changes in any geographical region. Open‑source, high‑resolution Google Earth satellite imagery (Maxar Technologies) with 30 cm resolution from 2006 and 2019 were used to examine the spread of asbestos. In the GIS analysis, these visible asbestos‑spread regions were carefully digitized using ArcMAP 10.8 version software (Environmental Systems Research Institute [ESRI]), and the change in area was estimated to be from 2006 to 2019.

The study was approved by the institutional ethics committee. There was no external funding support.

## Results

### Soil sample analysis

**Figure 3:**
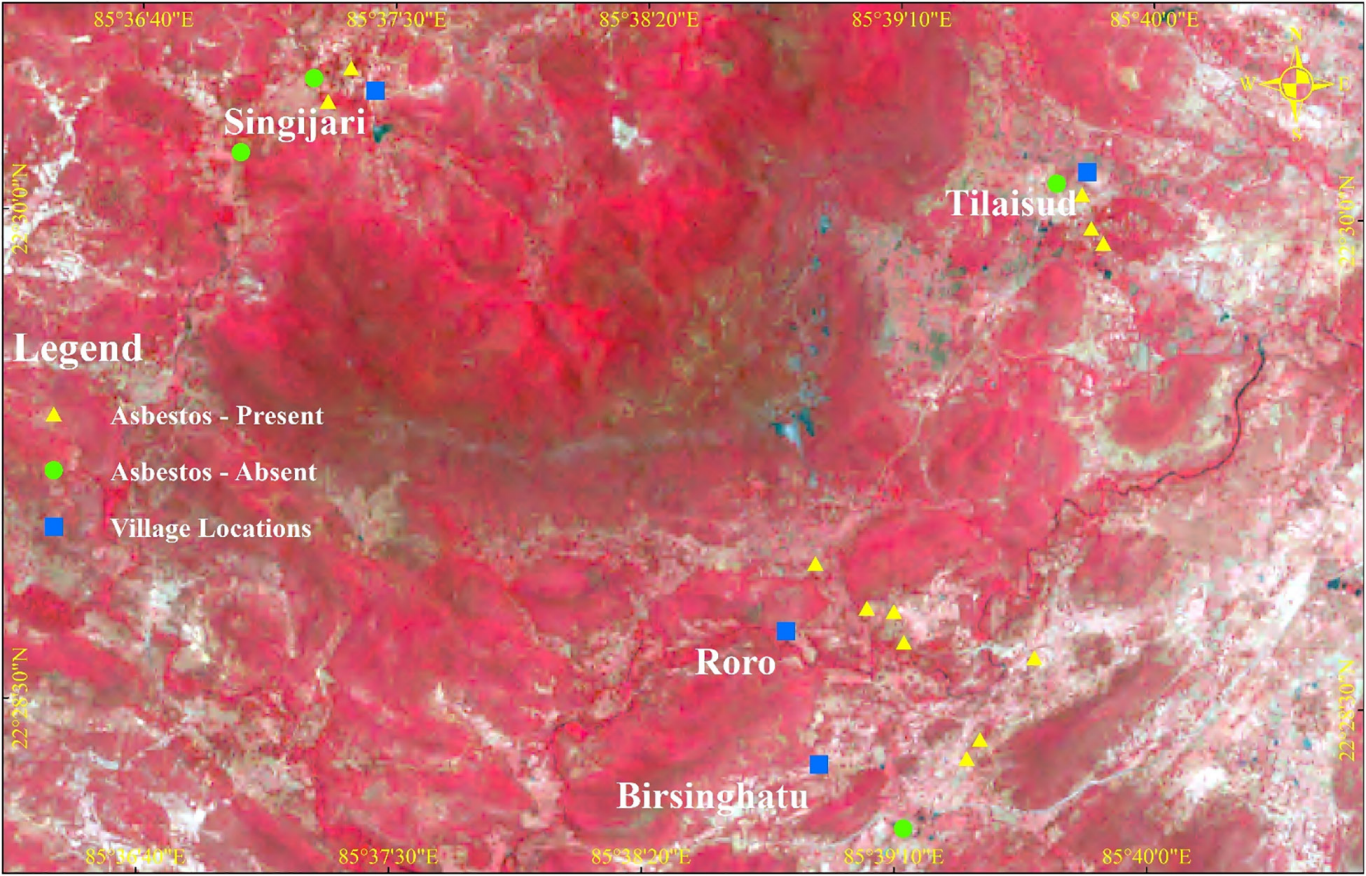
Soil sample locations in villages around the mine.

The laboratory gave results of all the soil samples, including one negative control and 16 test samples from study villages. Of the 16 samples, 12 were found to be positive for asbestos. All villages had some positive samples. The geographical locations of all positive and negative samples are given in Figure 3. The locations for positive samples are indicated by yellow triangles and the negative samples by green dots.

Table 1 summarizes the results of soil sample testing from the laboratory. In every village, at least two (50%) of the four samples were positive. All positive samples contained chrysotile asbestos with long fibers. The fiber lengths were much longer compared with other asbestos types from the amphibole family. Long asbestos fibers (LAF) are more carcinogenic and fibrogenic, as some propose [16].

**Table 1:** Soil sample location with results for asbestos testing.

SAMPLE NUMBER	VILLAGE	COORDINATES OF SAMPLE LOCATION		PRESENCE OF ASBESTOS (Y/N)	TYPE OF ASBESTOS	FIBER TYPE (LAF/SAF)
		LATITUDE	LONGITUDE			
1.	Tilaisud	22.501751	85.661413	No	—	
2.	Tilaisud	22.501200	85.662803	**Yes**	Chrysotile	LAF
3.	Tilaisud	22.499486	85.663327	**Yes**	Chrysotile	LAF
4.	Tilaisud	22.499171	85.663658	**Yes**	Chrysotile	LAF
5.	Roro	22.482237	85.648328	**Yes**	Chrysotile	LAF
6.	Roro	22.548490	85.796351	**Yes**	Chrysotile	LAF
7.	Roro	22.479828	85.652669	**Yes**	Chrysotile	LAF
8.	Roro	22.478254	85.653222	**Yes**	Chrysotile	LAF
9.	Birsingh Hatu	22.472362	85.656727	**Yes**	Chrysotile	LAF
10.	Birsingh Hatu	22.548604	85.796713	**Yes**	Chrysotile	LAF
11.	Birsingh Hatu	22.548529	85.795499	**Yes**	Chrysotile	LAF
12.	Birsingh Hatu	22.548333	85.796530	No	—	
13.	Singijari	22.550501	85.805749	No	—	
14.	Singijari	22.550132	85.805201	**Yes**	Chrysotile	LAF
15.	Singijari	22.550127	85.805100	**Yes**	Chrysotile	LAF
16.	Singijari	22.550172	85.805201	No	—	

**Figure 4:**
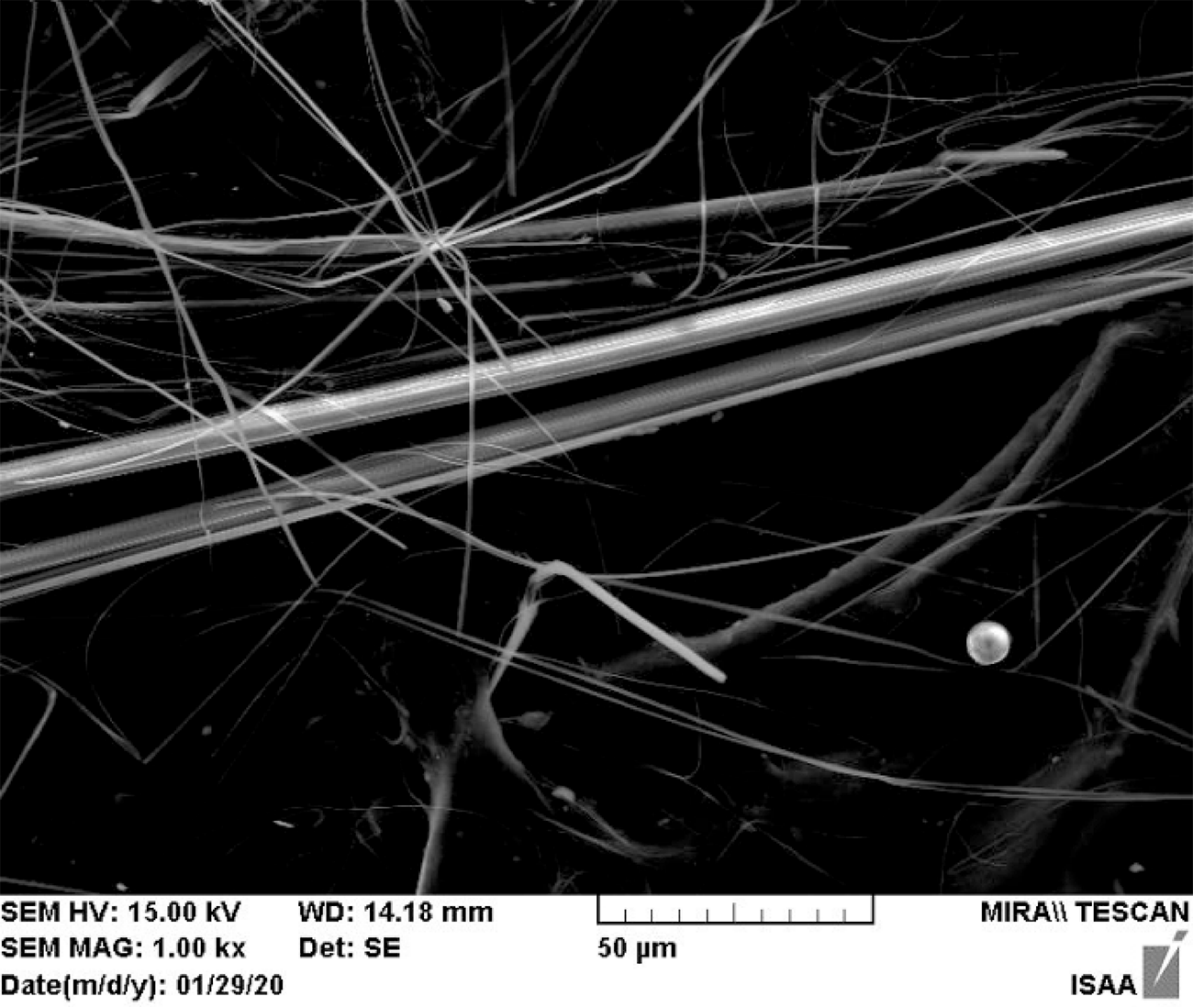
Asbestos fiber from sample 3 as seen under an electronic microscope.

**Figure 5:**
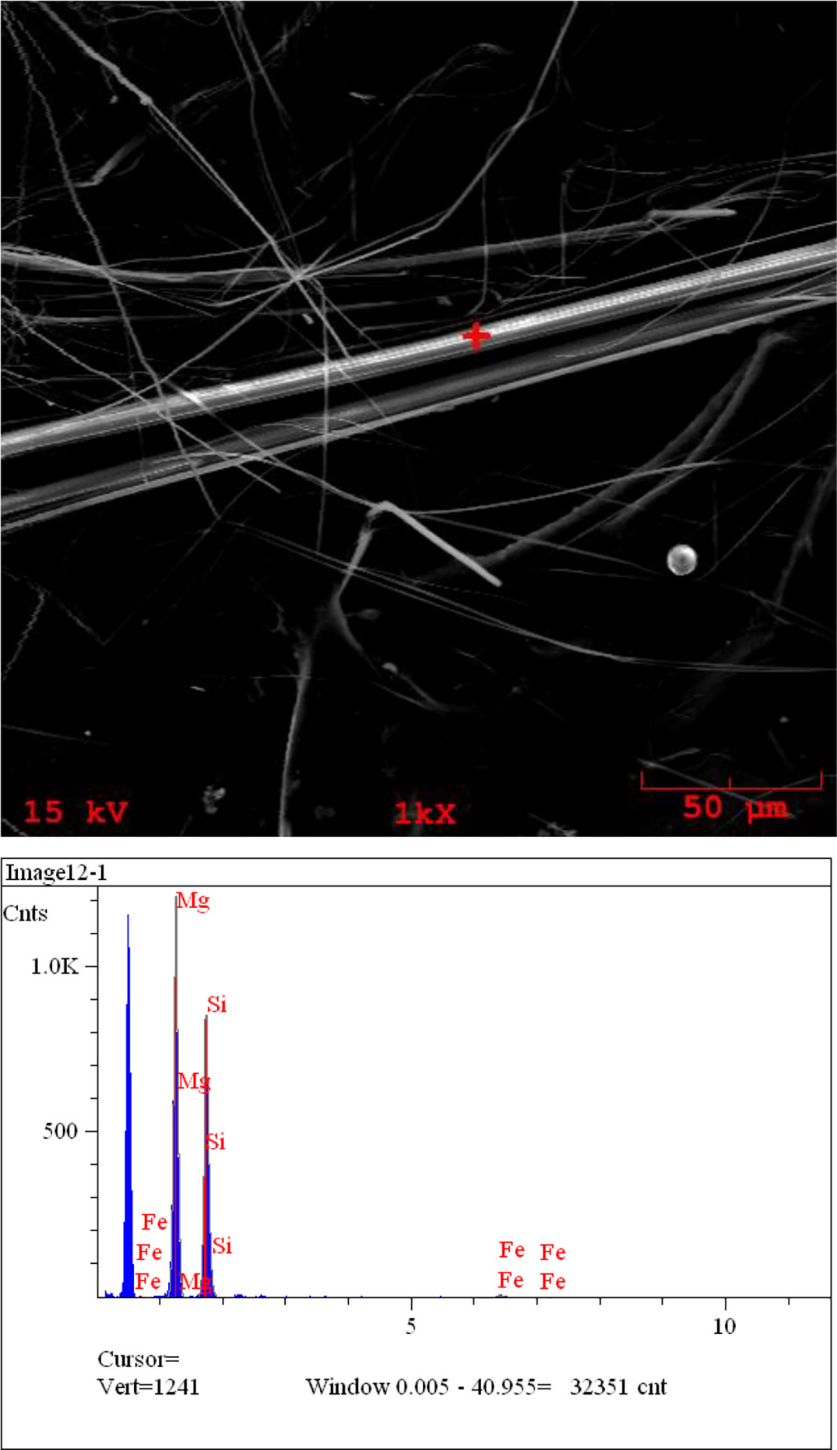
Image of asbestos fiber under an electron microscope and chemical analysis of the fiber with element composition at the marked point of the fiber.

The laboratory provided electron microscopic photos of the samples with asbestos fibers and their atomic composition graph. This graph is used to identify asbestos and its sub‑types. As an example, the results of one positive sample (sample number 2) are elaborated here. Figure 4 gives a picture of the identified asbestos fiber, and Figure 5 indicates the location of the fiber that was analyzed for its chemical composition. Scanning electron microscopy (SEM) can get the atomic composition of the materials at that dot. The graph indicates the number of atoms of various elements. This also gives a proportion of atoms of the elements present. This proportion is given in Table 2 for the sample in this example. This proportion helps to determine the type of asbestos.

**Table 2:** The table from the report of soil test results for sample 2, giving the element composition of the identified fiber, which helps identify it as chrysotile asbestos.

ELEMENT	LINE	ATOMIC PERCENTAGE (%)	ATOMIC RATIO	CONCENTRATION (WT%)
Mg	Ka	50.713	1.0000	46.584
Si	Ka	48.240	0.9512	51.205
Fe	Ka	1.047	0.0207	2.210
		100.000 (Total)		100 (Total)

## Map Analysis for the Change Detection of Asbestos Spread

**Figure 6:**
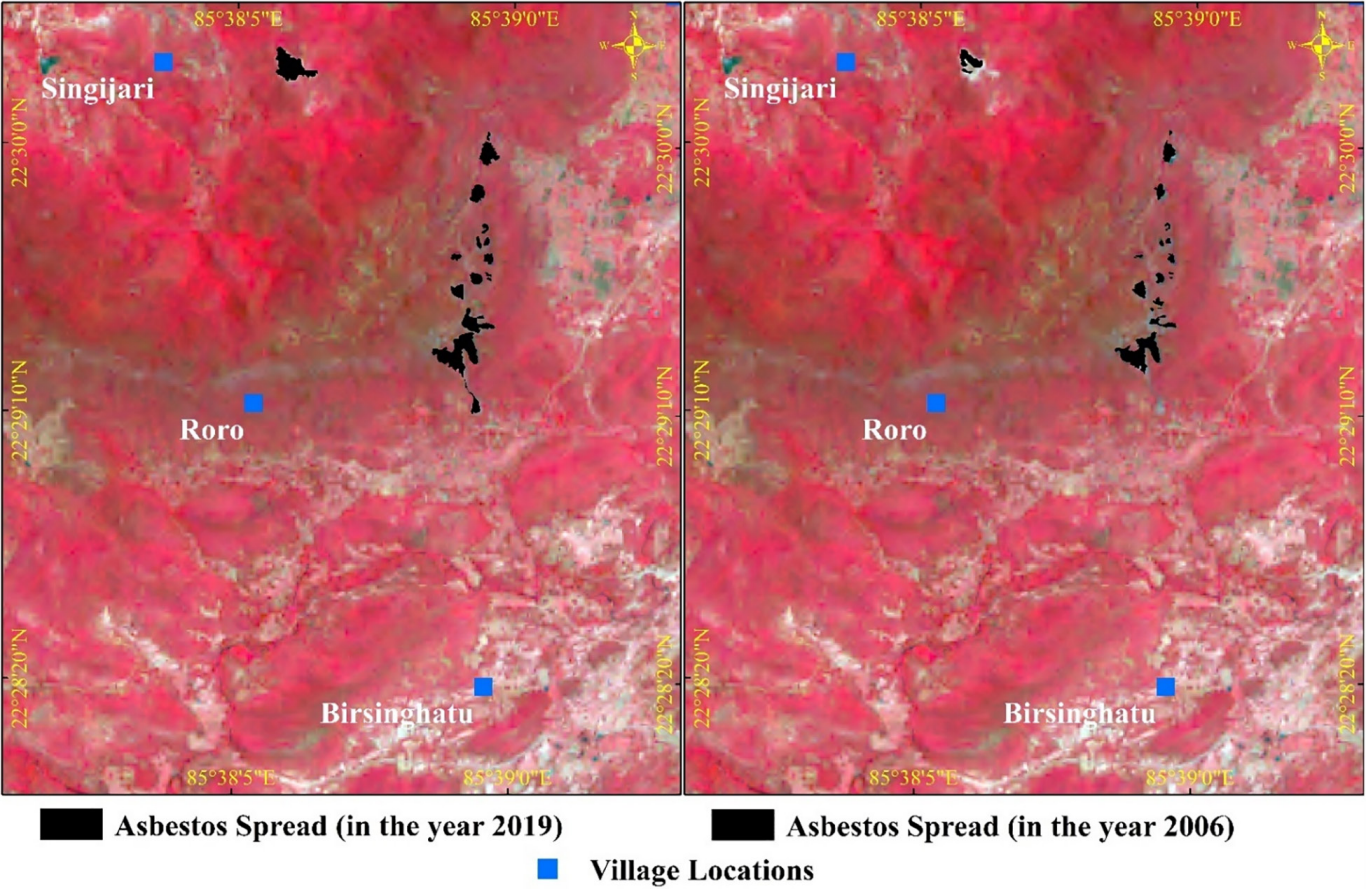
Area of asbestos spread analyzed using temporal Google Earth satellite imageries and super imposed over the FCC of Sentinel 2 images.

The areas mapped out using satellite imagery were compared. Before 2006, such high‑resolution images of this area were unavailable in the public domain. The analysis was limited to the asbestos dump near the mine site only. The visible dump sites were carefully marked in both images. These were the same locations with varying areas of asbestos spread. In Figure 6, the asbestos dumps can be seen as black‑plotted areas, which were identified using Google Earth satellite images superimposed over the false color composite (FCC) of the European Space Agency’s Sentinel 2 satellite data with 10 m resolution. The comparative analysis showed an increase in the asbestos dump area from 83,366.01 m^2^ in 2006 to 100,105.88 m^2^ in 2019, indicating a net increase by 16,739 m^2^ (20.08%) in the area in this period. That means the area increased by around 20% in those 13 years, indicating that, due to natural forces (e.g., wind and rain), the powdery asbestos dump located around the mine site was spreading.

This spread was calculated for the visibly identifiable asbestos‑contaminated areas. However, the contamination of soils by asbestos in minute concentrations cannot be captured through GIS images but rather requires quantitative soil sample testing. Such spread in small quantities may be more distant and equally dangerous. In visibly normal village areas, detectable levels of asbestos are found in soil sample testing as presented above in the first component of the study.

## Discussion

The soil in residential areas was contaminated with asbestos, and the residents were in ongoing contact with it. Residents in all four study villages surrounding the Roro mine were exposed to asbestos in the soil. It has been established that contaminated soil can expose people while doing routine activities [7, 8]. Usual residents are generally unaware of their asbestos exposure and its possible health effects.

The findings from this study are relevant because little is known about post‑closure exposure to asbestos and the possibility of ARDs around abandoned Indian mines.

Community exposure to asbestos appears to be increasing for the villagers. Asbestos exposure and ARDs are commonly viewed from an occupational perspective. However, recent evidence shows that environmentally exposed community members in the mining area are at a higher risk for ARDs [7, 8]. The present study provides evidence of environmental exposure around an abandoned asbestos mine and points to the possibility of a similar situation at other mining locations. There are studies from other countries that establish a link between ambient asbestos exposure and ARDs [17–22]. That indicates a possible risk of ARDs for the residents of Roro Valley.

This study established that the area of contamination is increasing with time, although the mine was closed decades ago. If the grossly visible area of asbestos contamination is increasing this quickly, it is likely that the invisible dust is spreading in the vicinity of the mine. A study from Brazil showed that ARD‑related mortality rates were significantly higher for the geographies processing asbestos, suggesting a higher risk for exposure and ARDs at those locations [23]. Asbestos mines are often not very deep, unlike many other mines, such as coal mines. They are relatively closer to the surface, and many asbestos mines are of an open type (unlike Roro), and in those cases the possibility of asbestos spread is much higher. Mills and factories that process asbestos are also a source of contamination and exposure. One study from Rajasthan, India, indicated high concentrations (2.00–5.09 f/cm^3^ and 4.07–15.60 f/cm^3^) of asbestos fiber in the air around asbestos mills, and 30–40% of fibers were LAF [24]. The presence of asbestos in the environment even at such low concentrations is a serious health risk [3, 18, 19]. Recent studies from other countries indicate a higher occurrence of ARDs in the areas in the vicinity of asbestos mines, including lung cancer and mesothelioma [20, 21, 23, 25, 26].

The findings of this study highlight the importance of proper implementation of the laws related to the closure of the mines. The mine was not closed properly, its waste material was not managed as per the law, and there was no remediation of the peri‑mining area. Corporate responsibility for the environment and environmental health is not a major focus in India. There are many efforts in high‑income countries to contain environmental asbestos and prevent further exposure [27, 28]. Similar efforts are needed in India.

The study points toward the possibility that an improperly closed asbestos mine is likely a potential threat to human health and life unless the surrounding area is properly remediated. WHO and the International Labour Organization (ILO) have clearly stated that asbestos should be completely banned, and there is no safe way to handle it [3, 28].

Stringent laws should be enacted for the proper closure of all asbestos mines; otherwise, exposures will continue. Policies from developed countries provide good examples to emulate. However, there are some important differences in the Indian situation. The exposure risk here is likely to be higher due in part to inadequate implementation of the law. Awareness and access to public health are low for these interior tribal communities. Hence, related interventions and policies need to be considerate of vulnerable populations. Many researchers and research articles have flagged the burden and future risk of ARDs in India and the urgent need to improve related policies [28–32]. Unfortunately, in most LMICs, including India, asbestos exposure is not perceived as much of a threat in the larger public health discourse [33, 34].

Just as in most HICs, India should completely ban asbestos. However, even after the ban, closed mines may act as a potential source of exposure and ARDs. It is important that peri‑mine areas need to be studied and intervened in scientifically to stop ongoing and future asbestos exposure. All countries engaged in asbestos mining need to invest in environmental risk mapping and hazard mitigation in their respective locations.

## Data Availability

Data related to this manuscript will be available in the supplementary files. All authors had access to the data and played a role in writing the manuscript.
